# Supplementary material for: Getting More Out of Biomedical Documents with GATE's Full Lifecycle Open Source Text Analytics
Source: PLoS Comput Biol. 2013 Feb 7;9(2):e1002854. doi: 10.1371/journal.pcbi.1002854 (PMC3567135; doi:10.1371/journal.pcbi.1002854)
Supplement: Dataset S1 — GATE software. Dataset S1 bundled with this paper contains a distribution of GATE (or see http://gate.ac.uk/download/). (TGZ) [file pcbi.1002854.s001.tgz › plos-gate/doc/kiss.html]

GATE2 design philosophy


# It's so smart to be simple

From the Grauniad,
Thursday December 2, 1999.

Jack Schofield meets a critic who
gets paid $10,000 a day to tell
designers not to be too clever.

For only $10,000 a day, usability expert
Jakob Nielsen will tell you why your
website is rubbish. Surprisingly, what you
get for the money isn't a high-powered
marketing executive, or even an expert in
web programming. He's just a deliberately
ordinary user with a dial-up modem.

What's unusual about Nielsen is his
understanding of the way websites work -
and the growth of e-commerce has
suddenly made that valuable to lots of big
companies. As Nielsen says: "For
anybody who is selling online, even the
smallest usability improvement has an
incredibly positive pay-off ratio. The cost
of getting my feedback is much lower than
the benefit from taking my advice."

This idea has already been accepted in
the design world, partly through the work
of Donald Norman, the former Apple
usability expert who wrote The Design Of
Everyday Things and a partner in the
Nielsen Norman Group, a consultancy.

Nielsen, settling into a sofa in a central
London hotel, explains that usability is
even more important on the web than
businesses have realised, because
e-commerce has changed the way it
works.

"The web is no longer about brand image,
the web is about customer experience,"
Nielsen says. "The web reverses the
relationship between the two different
types of communication.

"In the industrial age, you got a huge
number of marketing impressions
compared with the number of
experiences. How often do you rent a car
compared with how often you see
television advertising or logos for car rental
companies? The web is the opposite: the
web is nothing but customer experience."
If the experience isn't good, users will go
to a different site to rent that car, or
whatever.

Nielsen cites American bookseller Barnes
and Noble as an example of a company
making this industrial age marketing
mistake on its website. "They weren't
focusing on how to buy a book, they were
focusing on building their brand, which is
so arrogant. They were saying: 'Because
we are a famous bookstore, you should
buy from us and not [Amazon].'
Everybody who tried the two sites decided
it was enormously easier to buy the book
from Amazon, so that's what they did."

Focus on the customer experience - how
easy is it to find and buy a book? - makes
it hard to justify the "bouncing heads,
spinning logos," and other graphics added
to make websites look cool.

"It's sad because animation is almost a
negative design element," Nielsen says.
"On the web today, animated graphics
equals useless in many people's minds,
so they ignore things even if they could in
fact be quite good. They skip the slogans,
they skip the branding messages, and
they skip the advertisements, so the
amount that goes into the user experience
is very very small."

Since users mentally strip out the rubbish
there's not much point in putting it in, and
I suggest Google, the search engine at
www.google.com, as a wonderful example
of minimalist design.

"That's truly one of my favourites," says
Nielsen, "but I didn't want to mention it
because I've been involved with them. But
the real message of Google is that the
other search engines have abandoned
their responsibilities. It's a disgrace,
really. They got so caught up in chasing
the latest fad they forgot why people go
there. Every time they do a redesign their
search boxes get smaller and smaller and
harder to find. They're spiting their
customers, really. In the long term, that's
not a viable strategy.

"Google has focused on taking one thing
that we know is really important -
searching - and doing it much better than
the others. The others are just second
rate now, so that's where they should
focus."

Another advantage of minimalist designs
is that they can be fast. "Response times
rule the web: if it's faster, it's better,"
Nielsen exclaims. "We say this because
when we do studies, all users say the
same thing: they don't want to wait for
slow download times.

"Other people did research on [computer]
response times back in the 1960s, and
we know they have to be less than one
second. Subsecond response times
dramatically enhance the user's feeling of
confidence and control and of under
standing what they're doing. Productivity
goes way up as well, because now you
dare to move where you want to move.
When the user worries - 'should I click or
should I not click? It's going to hurt me if I
do' - you get a suboptimal use of the
system.

"We've known this for 30 years, so why
don't people follow it? The fact that it's
based on a different technology doesn't
matter, because we're talking about
humans here and they're the same...
unless you give everyone a lobotomy or
something to slow them down."

Nielsen's own website, Alertbox, is not
just minimalist, it looks almost
undesigned. I ask Nielsen if it puts people
off because it's so clearly intended to be
not flash?

"That's true: it's almost like a provocation
that I do it the way I do," he says. "I would
not necessarily recommend that, say, an
e-commerce site be done in that style.
But I do want to emphasise that you can
do websites based on text. There's so
much under-emphasis on content on the
web today, but it's why people are online
to begin with.

"There are cases where it's appropriate to
do a video or an animation, and as we get
more bandwidth, more places will emerge
where it's appropriate to use multimedia.
However, it's almost unheard of to see
good uses of multimedia on the web
today. It's almost always used for effect,
not for something users benefit from.

"I'm the tireless defender of users' rights,
and in the modern world, there's a very
great degree of correspondence between
what's right for users and what companies
need to do to survive. You can't just
design what you like, you've got to design
what users like.

"And if you don't do it, someone else will.
There are millions of websites out there
and people will go where they're well
treated. You've got to tell people that
ultimate truth, even if it's very difficult to
do."

Jakob Nielsen publishes his articles
about website design free at
www.alertbox.com.
Nielsen Norman Group is at
www.nngroup.com
